# Bilateral Carotid Artery Molecular Calcification Assessed by [^18^F] Fluoride PET/CT: Correlation with Cardiovascular and Thromboembolic Risk Factors

**DOI:** 10.3390/life13102070

**Published:** 2023-10-17

**Authors:** Shiv Patil, Eric M. Teichner, Robert C. Subtirelu, Chitra Parikh, Omar Al-Daoud, Miraziz Ismoilov, Thomas Werner, Poul Flemming Høilund-Carlsen, Abass Alavi

**Affiliations:** 1 Sidney Kimmel Medical College, Thomas Jefferson University, Philadelphia, PA 19107, USA; shiv.patil@students.jefferson.edu (S.P.); eric.teichner@gmail.com (E.M.T.); chitra.parikh@students.jefferson.edu (C.P.); 2 Department of Radiology, Hospital of the University of Pennsylvania, Philadelphia, PA 390111, USA; robert.subtirelu@pennmedicine.upenn.edu (R.C.S.); omar.al-daoud@pennmedicine.upenn.edu (O.A.-D.); miraziz.ismoilov@pennmedicine.upenn.edu (M.I.); tom.werner@pennmedicine.upenn.edu (T.W.); 3 Department of Nuclear Medicine, Odense University Hospital, 5000 Odense, Denmark; pfhc@rsyd.dk; 4 Department of Clinical Research, University of Southern Denmark, 5230 Odense, Denmark

**Keywords:** aging, PET/CT, [^18^F] NaF PET/CT, cardiovascular risk, quantitative analysis

## Abstract

Atherosclerosis, a leading cause of mortality and morbidity worldwide, involves inflammatory processes that result in plaque formation and calcification. The early detection of the molecular changes underlying these processes is crucial for effective disease management. This study utilized positron emission tomography/computed tomography (PET/CT) with [^18^F] sodium fluoride (NaF) as a tracer to visualize active calcification and inflammation at the molecular level. Our aim was to investigate the association between cardiovascular risk factors and [^18^F] NaF uptake in the left and right common carotid arteries (LCC and RCC). A cohort of 102 subjects, comprising both at-risk individuals and healthy controls, underwent [^18^F] NaF PET/CT imaging. The results revealed significant correlations between [^18^F] NaF uptake and cardiovascular risk factors such as age (β = 0.005, 95% CI 0.003–0.008, *p* < 0.01 in LCC and β = 0.006, 95% CI 0.004–0.009, *p* < 0.01 in RCC), male gender (β = −0.08, 95% CI −0.173–−0.002, *p* = 0.04 in LCC and β = −0.13, 95% CI −0.21–−0.06, *p* < 0.01 in RCC), BMI (β = 0.02, 95% CI 0.01–0.03, *p* < 0.01 in LCC and β = 0.02, 95% CI 0.01–0.03, *p* < 0.01 in RCC), fibrinogen (β = 0.006, 95% CI 0.0009–0.01, *p* = 0.02 in LCC and β = 0.005, 95% CI 0.001–0.01, *p* = 0.01), HDL cholesterol (β = 0.13, 95% CI 0.04–0.21, *p* < 0.01 in RCC only), and CRP (β = −0.01, 95% CI −0.02–0.001, *p* = 0.03 in RCC only). Subjects at risk showed a higher [^18^F] NaF uptake compared to healthy controls (one-way ANOVA; *p* = 0.02 in LCC and *p* = 0.04 in RCC), and uptake increased with estimated cardiovascular risk (one-way ANOVA, *p* < 0.01 in LCC only). These findings underscore the potential of [^18^F] NaF PET/CT as a sensitive tool for the early detection of atherosclerotic plaque, assessment of cardiovascular risk, and monitoring of disease progression. Further research is needed to validate the technique’s predictive value and its potential impact on clinical outcomes.

## 1. Introduction

Atherosclerosis is the leading cause of death and disability among adults worldwide [1]. Atherosclerotic plaque formation involves inflammatory processes that can cause a number of complications, such as plaque rupture, erosion, and calcified nodule formation [2]. From a pathophysiological standpoint, it is believed that plaque formation begins, typically, due to endothelial cell dysfunction [3]. The associated following changes, leading to plaque calcification over time, are a major risk factor for cardiovascular disease (CVD) such as stroke and vascular dementia [4,5,6].

Computed tomography (CT) imaging and echocardiography are commonly employed for visualizing atherosclerosis, offering a precise delineation of cardiac structure and function [7,8]. However, these structural imaging techniques fall short in assessing key molecular changes, especially during the early stages of atherosclerotic plaque formation [9]. Additionally, they may lack the resolution necessary to detect microcalcifications [10]. Early detection of the molecular alterations that drive plaque formation holds substantial clinical value for halting disease progression, either through lifestyle modifications or pharmacological management of associated comorbidities [11]. The early identification of molecular markers for calcification and inflammation is likewise crucial for predicting subsequent changes, such as reductions in perfusion and tissue viability [12]. In this context, the employment of sensitive and specific molecular imaging techniques for visualizing plaque formation and assessing vascular integrity—across the entire disease trajectory—could potentially enhance both the management and outcomes for individual patients. Considering that cardiovascular diseases (CVDs) are a leading cause of morbidity and mortality worldwide, refining techniques to monitor disease progression and implement timely interventions could exert a profound impact on overall patient health [13,14,15].

Positron emission tomography (PET) serves as a molecular imaging technique capable of detecting active molecular processes, such as early arterial calcification, prior to the manifestation of structural changes. The hybrid technology of PET and computed tomography (PET/CT) offers an enhanced spatial resolution, along with the capability to visualize molecular alterations throughout the progression of atherosclerosis [16]. The tracers most frequently utilized for this purpose are [^18^F] fluorodeoxyglucose (FDG) and [^18^F] sodium fluoride (NaF), which serve as indicators of inflammation and active microcalcification, respectively [17,18]. FDG-PET/CT has previously been correlated with carotid artery inflammation, which cannot be predicted by structural changes alone [19]. The uptake of [^18^F] NaF is deemed clinically significant in atherosclerosis, acting as a potential prognostic marker for cardiovascular events linked to microcalcification, such as myocardial infarction, angina, and coronary artery disease [20]. Elucidating the relationship between [^18^F] NaF uptake and arterial calcification could provide valuable insights for clinicians, informing their assessment of cardiovascular risk.

It is worth noting that [^18^F] NaF uptake is primarily influenced by factors such as the injected dose, blood activity, and the specific PET/CT system utilized [21]. One distinct advantage of [^18^F] NaF is its ability to uniquely identify abnormal uptake patterns, including vascular microcalcification associated with coronary artery disease, independent of physiologic myocardial activity [12,22]. In contrast, such abnormal uptake is physiologically masked in FDG-PET/CT imaging, necessitating additional patient preparation to accurately identify alterations in disease state [23]. While various protocols exist for patient preparation in FDG-PET/CT imaging, these commonly center on dietary modifications and/or fasting periods prior to the scan [24].

Previous work by Dweck et al. has demonstrated increased [^18^F] NaF uptake in the coronary arteries among patients possessing high-risk cardiovascular profiles [25]. Subsequent studies have corroborated the relationship between [^18^F] NaF uptake and cardiovascular risk factors in other vascular regions, thus endorsing the utility of [^18^F] NaF in evaluating cardiovascular detriment [26,27,28] The tracer also holds potential for monitoring the effectiveness of therapeutic interventions [29]. Nevertheless, data concerning the common carotid arteries remain sparse. In the present study, we aim to identify associations between cardiovascular risk and molecular calcification in both the left common carotid artery (LCC) and the right common carotid artery (RCC) using [^18^F] NaF.

## 2. Methods

### 2.1. Study Population

A total of 102 individuals were selected from a total of 139 participants enrolled in the Cardiovascular Molecular Calcification Assessed by [^18^F] NaF PET/CT (CAMONA) study. For each participant, the criterion for selection was defined by whether the quality of the PET/CT image was sufficient to allow for precise LCC and RCC segmentation. Indication for receiving PET/CT was defined by eligibility for CACS (coronary artery calcium scoring) due to symptoms suggesting angina pectoris. This study received approval from the Danish National Committee on Health Research Ethics and is registered on ClinicalTrials.gov under the identifier NCT01724749, aligning with the principles outlined in the Declaration of Helsinki. Prior to their participation in the study, all participants provided written informed consent.

### 2.2. Patient Evaluation

Of the 102 subjects, 38 individuals were considered at-risk for CVD and 64 were healthy controls. Healthy controls were recruited from a random sample of Danish citizens without prior history or symptoms of CVD. Individuals were identified as being at risk for CVD development based on risk stratification algorithms such as the Framingham Risk Score (FRS), which estimates the risk of heart attack within 10 years, and the European SCORE system, which predicts the 10-year risk of cardiovascular death based on factors such as gender, smoking status, age, systolic blood pressure, and cholesterol levels [30,31,32]. Individuals who were not using any blood pressure medications and who had a greater than 1% increased risk of fatal CVD, estimated using the SCORE tool, were eligible for inclusion into the at-risk group [33]. Certain patients were excluded from the study due to factors such as a history of pregnancy, malignancy within the past 5 years, known immunodeficiency, history of deep vein thrombosis or pulmonary embolism within the prior 3 months, alcohol or illicit drug use/abuse, mental illness, and statin therapy use.

Physical examinations were conducted for each participant to rule out any evident signs of atherosclerotic disease. Blood samples were collected to assess white blood cell (WBC) count, lipid profile, fasting plasma glucose, glycated hemoglobin (HbA1c), C-reactive protein (CRP), homocysteine, fibrinogen, and creatinine levels. Office blood pressure readings were taken, with individuals being classified as hypertensive if their average systolic pressure exceeded 120 mmHg and their average diastolic pressure exceeded 80 mmHg. Renal function was determined from the calculation of the glomerular filtration rate using the Chronic Kidney Disease Epidemiology Collaboration equation, as previously described [33]. Each participant was evaluated for their smoking habits and alcohol intake. Additionally, body mass index (BMI) was measured for all subjects.

### 2.3. Cardiac PET/CT Acquisition Protocol

[^18^F] NaF PET/CT scans were carried out based on the guidelines set by the CAMONA research team [8,16]. Using hybrid PET/CT equipment, namely GE Discovery STE, VCT, RX, and 690/710 models, the hospital’s scheduling team randomly assigned a scanner to each individual. The [^18^F] NaF PET/CT scan was initiated 90 min after administering an intravenous dose of 2.2 MBq of [^18^F] NaF for each kilogram of the individual’s weight. Adjustments were made to the PET images for factors such as attenuation, scatter, random events, and inactivity periods of the scanner. For the reconstruction of the PET data, the Ordered Subset Expectation Maximization (OSEM) approach was employed. Moreover, to aid in attenuation adjustments and delineate anatomical landmarks, low-intensity CT scans (140 kV, 30–110 mA, noise rate at 25, 0.8 s for each turn, with a slice measurement of 3.75 mm) were taken. The overall radiation exposure from the entire scanning procedure was around 6.7 mSv.

### 2.4. Carotid PET/CT Data Analysis

**Figure 1:**
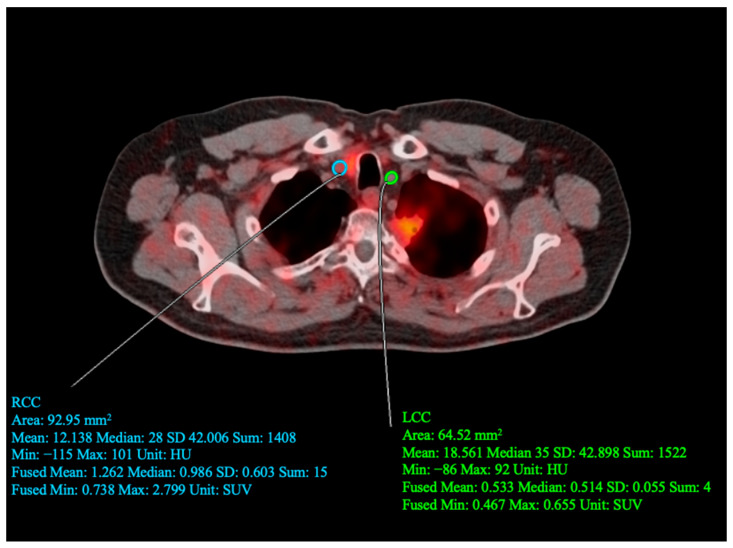
Sample of quantitative assessment conducted by drawing regions of interest (ROI) around the left (LCC) and right (RCC) common carotid arteries. For the drawn RCC ROI: SUVmean = 1.262, SUVmin = 0.0738, and SUVmax = 2.799. For the drawn LCC ROI: SUVmean = 0.533, SUVmin = 0.467, and SUVmax = 0.655.

Quantitative analysis of the fused PET/CT images was conducted using OsiriX 7.5.1 software (Pixmeo SARL), specifically to determine the average SUVmax (aSUVmax). A region of interest (ROI) was first manually delineated on the fused axial image encompassing either the LCC or RCC. The ROI spanned the whole structure with a slice thickness of 3.75 mm. Measurements of SUVmax were carried out on PET images that had been corrected for CT attenuation. To calculate aSUVmax, the uncorrected SUVmax values for each individual slice were recorded, summed, and divided by the total number of slices (Figure 1). To account for potential inaccuracies due to spillover activity from nearby [^18^F] NaF-avid anatomical areas, blood-pool correction was applied by measuring the activity exclusively in the inferior vena cava. This approach was consistent with the methodology employed by prior authors of the CAMONA study.

### 2.5. Statistical Analysis

Continuous variables were presented either as mean ± SD in cases of normal distribution or as median (25th–75th percentile) when not normally distributed. To assess normality, the one-sample Kolmogorov–Smirnov test was applied to all continuous variables. Statistical comparisons for continuous variables were conducted using an independent one-way ANOVA test. Categorical data were represented in either counts or percentages. The chi-squared test or Fisher’s exact test was utilized for analysis of categorical data when appropriate. Correlations between LCC and RCC [^18^F] NaF uptake and baseline covariates were determined through multivariable linear regression analysis. Initially, all variables exhibiting a statistically significant association in univariate analysis were included in the multivariable model. If applicable, a stepwise backward elimination process was subsequently applied to remove variables with a *p*-value > 0.05, with the objective of retaining only those covariates essential for explaining variance while avoiding excessive complexity. To prevent overfitting, all potential confounding variables were initially introduced into the multivariable model based on their clinical relevance. Variables with a *p*-value > 0.20 were then excluded as determined by the log-likelihood test. The variance inflation factor was assessed to detect multicollinearity among covariates, with values exceeding 3.5 indicating potential multicollinearity. The Durbin–Watson test was performed to assess the independence of observations. Normality of residuals from linear regression was assessed using a normal probability plot. Statistical significance was considered at the α = 0.05 level. All analyses were performed using R version 4.0.3.

## 3. Results

### 3.1. Clinical Characteristics of the Study Population

The baseline characteristics of the study population are summarized in Table 1. In total, one hundred and two subjects were included in the analysis (mean age 48.4 ± 14 years; 52 (51%) males). Twelve (11%) patients were active smokers, 45 (44%) had arterial HTN, and 18 (17%) had hypercholesterolemia. History of peripheral artery disease and previous stroke or transient ischemic attack were reported in four (4%) and two (2%) patients, respectively. The mean BMI was 26.5 ± 4.0 kg/m^2^ with 48 (47%) being overweight (BMI 25–29.9) and 18 (17%) obese (BMI ≥ 30). The median 10-year FRS was 8% (2–10%).

### 3.2. Association between Cardiovascular Risk Factors and Arterial Molecular Calcification

**Figure 2:**
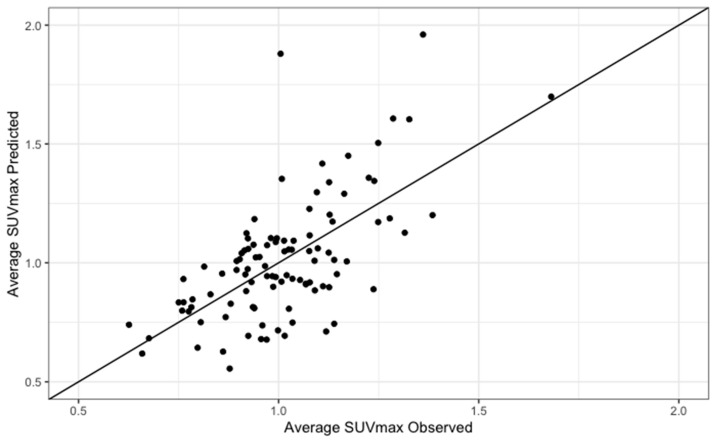
Predicted versus observed values of average SUV max for the LCC.

**Figure 3:**
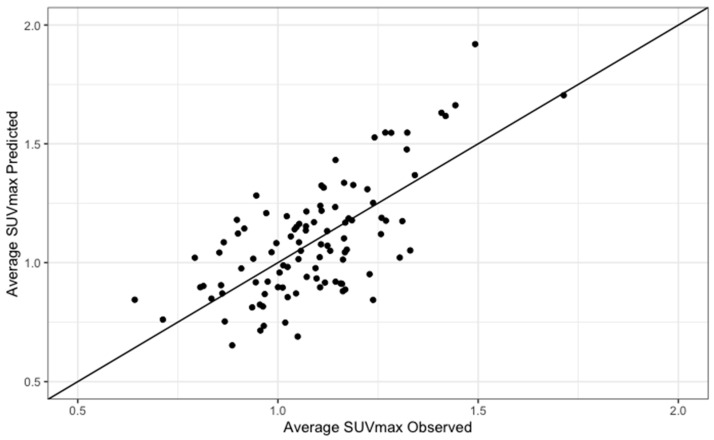
Predicted versus observed values of average SUV max for the RCC.

To determine whether baseline covariates could be significantly associated with LCC and RCC [^18^F] NaF uptake, multiple regression analyses were conducted as shown in Table 2 and Table 3. All variables that showed a statistically significant correlation at univariate analysis were entered into the multivariable model. While age, BMI, and fibrinogen correlated with both LCC and RCC aSUVmax, gender, high-density lipoprotein (HDL) cholesterol, and CRP significantly associated with RCC aSUVmax only. All covariates were used in the multivariable models for both LCC and RCC for consistency. Statistically significant regression equations were found for LCC (F (6, 95) = 11, *p* < 0.01, R^2^ = 0.38) and RCC (F (6, 95) = 15, *p* < 0.01, R^2^ = 0.46) (Figure 2 and Figure 3). Age (β = 0.005, 95% CI 0.003–0.008, *p* < 0.01), BMI (β = 0.02, 95% CI 0.01–0.03, *p* < 0.01), and fibrinogen (β = 0.006, 95% CI 0.0009–0.01, *p* = 0.02) all directly correlated with LCC aSUVmax. Male gender (β = −0.08, 95% CI −0.173–−0.002, *p* = 0.04) inversely correlated with LCC aSUVmax. For RCC aSUVmax, direct correlations were found with age (β = 0.006, 95% CI 0.004–0.009, *p* < 0.01), BMI (β = 0.02, 95% CI 0.01–0.03, *p* < 0.01), fibrinogen (β = 0.005, 95% CI 0.001–0.01, *p* = 0.01), and HDL cholesterol (β = 0.13, 95% CI 0.04–0.21, *p* < 0.01), while an inverse association was observed with male gender (β = −0.13, 95% CI −0.21––0.06, *p* < 0.01) and CRP (β = −0.01, 95% CI −0.02–0.001, *p* = 0.03). The final predictive model for LCC and RCC aSUVmax were the following:

LCC aSUVmax = −0.1 + (0.005*age) − (0.08*gender) + (0.02*BMI) + (0.07*HDL cholesterol) − (0.01*CRP) + (0.006*fibrinogen)

RCC aSUVmax = 0.008 + (0.006*age) − (0.13*gender) + (0.02*BMI) + (0.13*HDL cholesterol) − (0.01*CRP) + (0.005*fibrinogen),

where age is expressed in years, BMI in kg/m^2^, HDL cholesterol in mmol/L, CRP in mg/L, fibrinogen in μmol/L, and gender is coded as 1 = male or 0 = female.

### 3.3. Correlation between Cardiovascular Risk and Arterial Molecular Calcification

**Figure 4:**
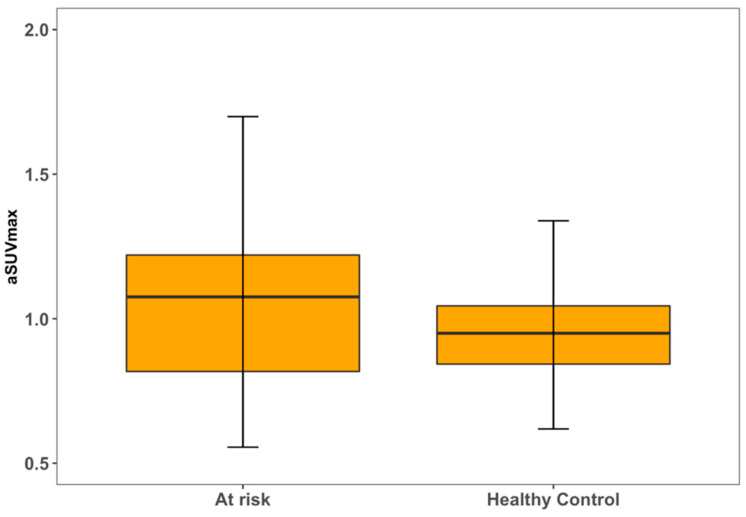
Left carotid artery [^18^F] NaF uptake in relation to disease status.

**Figure 5:**
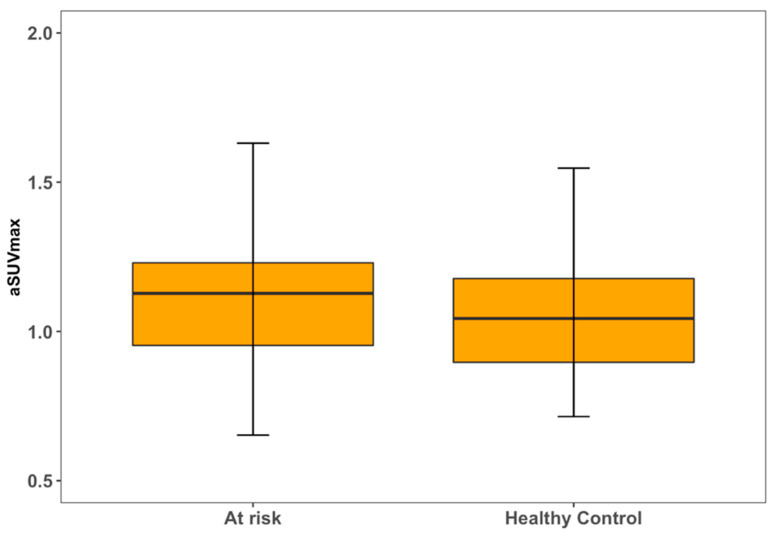
Right carotid artery [^18^F] NaF uptake in relation to disease status.

**Figure 6:**
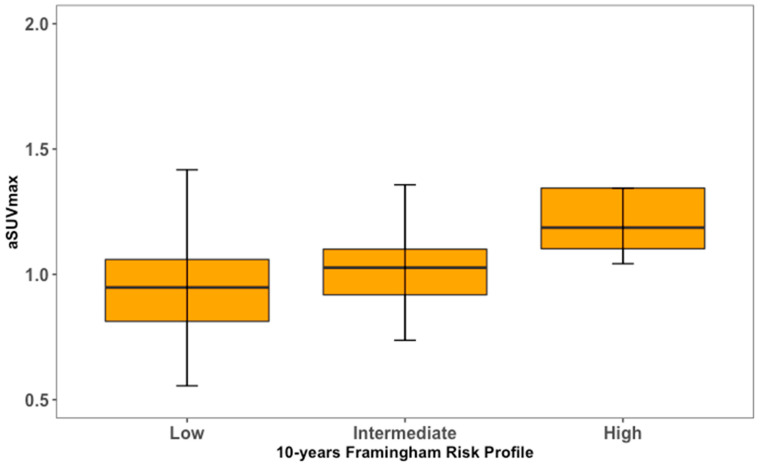
Left carotid artery [^18^F] NaF uptake in relation to cardiovascular risk profile.

**Figure 7:**
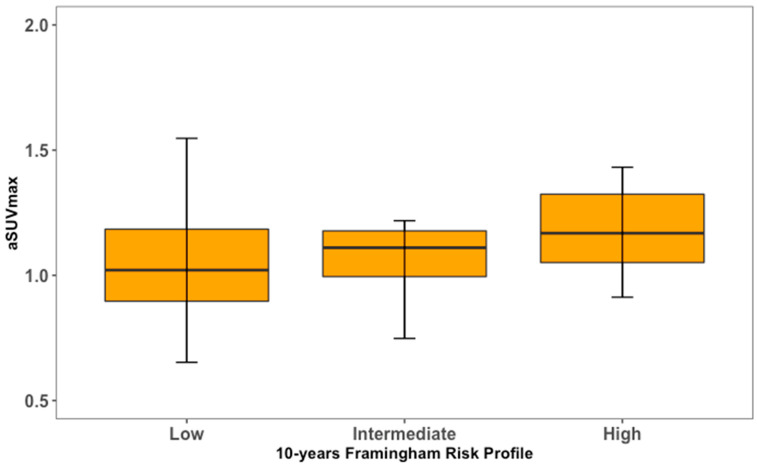
Right carotid artery [^18^F] NaF uptake in relation to cardiovascular risk profile.

For both LCC and RCC, aSUVmax was higher in at-risk patients compared to healthy controls (Figure 4 and Figure 5). LCC aSUVmax was 1.08 ± 0.33 for at-risk patients and 0.96 ± 0.19 for healthy controls (one-way ANOVA, *p* = 0.02). RCC aSUVmax was 1.14 ± 0.28 for at-risk patients and 1.04 ± 0.22 for healthy controls (one-way ANOVA, *p* = 0.04). The LCC aSUVmax also increased according to the 10-year risk of major adverse cardiovascular events estimated using the FRS (Figure 6). Individuals at low risk (<10%) had the lowest aSUVmax of 0.96 ± 0.22, those at intermediate risk (10–20%) had an aSUVmax of 1.03 ± 0.21, and high-risk individuals (≥20%) had the highest aSUVmax of 1.29 ± 0.39 (one-way ANOVA, *p* < 0.01). No significant differences were observed among FRS groups for RCC aSUVmax (*p* = 0.11) (Figure 7).

## 4. Discussion

In the present study, associations were identified between cardiovascular risk and arterial molecular calcification of the LCC and RCC as measured by [^18^F] NaF. The major findings are as follows: (1) [^18^F] NaF uptake directly correlates with atherosclerotic risk factors such as age, BMI, and fibrinogen for both LCC and RCC. In the RCC only, we observed a direct correlation with HDL cholesterol and indirect correlations with male gender and CRP. (2) LCC and RCC [^18^F] NaF uptake are significantly higher in at-risk patients compared to healthy controls. (3) [^18^F] NaF uptake in the LCC increases according to the estimated risk of cardiovascular events as assessed by FRS, whereas no correlation was found between cardiovascular risk profile and [^18^F] NaF uptake in the RCC.

Atherosclerotic plaques have traditionally been identified through clinical findings and abnormalities in structural imaging [34]. These approaches, however, are not effective in the detection of early stage disease. Additionally, structural imaging, such as CT or echocardiography, cannot distinguish sites of active calcium deposition, where vulnerable plaques are likely to arise, from chronic vascular calcification characteristic of stable disease [35,36]. This is, in part, due to limitations derived from the resolution of CT scanners [37,38]. CT remains limited in spatial resolution despite recent advances in improving imaging quality, such as increasing *z*-axis coverage and the utilization of faster rotation times to increase temporal resolution [39,40].

To address these limitations, [^18^F] NaF PET/CT offers a promising imaging modality for the early detection of atherosclerotic plaque formation by identifying changes at the molecular level. Following chemisorption, the ^18^F ion exchanges rapidly for the OH^-^ ion of hydroxyapatite to form fluorapatite. Since hydroxyapatite in macroscopic deposits is internalized, and [^18^F] NaF is unable to penetrate the crystalline mass, [^18^F] NaF binding is able to specifically detect new calcifying activity [41]. This is in contrast to [^18^F] FDG, which has been reported to have variable efficacy in the detection of atherosclerosis and in correlation with cardiovascular risk factors, particularly due to high physiological myocardial uptake [42,43]. The low background activity and high specificity of [^18^F] NaF has supported the use of this tracer as the superior molecular biomarker of atherosclerotic calcification.

The feasibility of [^18^F] NaF as a surrogate measure of atherosclerotic plaque formation has now been demonstrated in multiple human studies. Moreover, correlations between [^18^F] NaF uptake and cardiovascular risk factors have been established across multiple vascular beds such as the thoracic aorta, coronary, femoral, and common carotid arteries. Derlin et al. previously identified associations between [^18^F] NaF uptake in the common carotid arteries and cardiovascular risk in a large sample of neurologically asymptomatic oncologic patients (*n* = 266, mean age 66.1 ± 12.4 years) [28]. In another sample of oncologic patients, Morbelli et al. showed a significant relationship between [^18^F] NaF uptake in the carotid artery and FRS risk factors (e.g., age, diabetes, smoking, BMI, and systolic blood pressure) with the exception of BMI, while visible calcification in CT was dependent on age only (*n* = 80, mean age 65.3 ± 8.2 years) [44]. [^18^F] NaF carotid uptake has also been correlated with the incidence and severity of atherosclerotic complications: Quirce et al. reported a higher [^18^F] NaF uptake in symptomatic carotid plaques of patients investigated for recent cerebrovascular accident (CVA) compared to that in asymptomatic plaques (age range 50–83 years) [45]. The findings from the present study support and extend these results in a population encompassing both healthy adults and patients at risk for CVD. Additionally, the mean age of the study population is comparatively low to those of the aforementioned publications, which typically involved elderly individuals with advanced cardiovascular disease or oncologic patients whose outcomes may not be perfectly generalizable to noncancer patients.

Our research is a follow-up of the study conducted by Castro et al., who showed an increased [^18^F] NaF uptake in the LCC of patients with cardiovascular and thromboembolic risk factors (*n* = 128, mean age 48 ± 14 years) [46]. Specifically, Castro et al. demonstrated that age, BMI, arterial hypertension, and level of physical activity (LPA) were independent associations of LCC [^18^F] NaF uptake. The results from the present study largely support these findings, as LCC [^18^F] NaF uptake was observed to correlate with age, BMI, and fibrinogen. We further show correlations between RCC [^18^F] NaF uptake and cardiovascular risk factors, which also comprise age, BMI, and fibrinogen, but include a direct association with HDL cholesterol and an inverse correlation with gender and CRP as well. The differences we found in risk factor correlations between LCC and RCC is consistent with previous research from Luo et al., who suggest that the different anatomical origins of the two common carotid arteries may explain discrepancies in the various risk factors that correlate with [^18^F] NaF uptake in the LCC and RCC [47]. However, while Luo et al. claim that the LCC is more susceptible to biochemical indices, the findings from our study suggest that it is the RCC that more strongly correlates with biochemical indices in the blood such as fibrinogen, HDL cholesterol, and CRP.

Castro et al. also reported a higher [^18^F] NaF uptake with increased cardiovascular risk as estimated by the FRS. The FRS is a system used to estimate the 10-year risk of cardiovascular disease events and consists of age, gender, total cholesterol, HDL cholesterol, smoking habits, and systolic blood pressure. Our results validate these findings in both the LCC and RCC, providing support for the association between [^18^F] NaF uptake and cardiovascular risk irrespective of carotid sidedness. Indeed, previous research has suggested that other chronic diseases, such as diabetes and renal failure, are also associated with carotid artery calcification and stenosis [48,49]. Future studies may seek to understand correlations of [^18^F] NaF uptake with cardiovascular risk in patients with other chronic comorbidities.

The results of our study should be considered in the context of its limitations. First, this study is cross-sectional, which provides only a static cardiovascular risk profile that does not capture potential temporal changes in the [^18^F] NaF uptake of individual subjects. Longitudinal data can inform us of within-subject variations in the association of [^18^F] NaF uptake and cardiovascular risk factors, which would assess the utility of [^18^F] NaF as a marker of CVD over the course of atherosclerotic disease progression. Another limitation of our study is that while we have correlated [^18^F] NaF uptake in both LCC and RCC with cardiovascular risk, it is unknown if these results are related to the long-term incidence of major adverse cardiovascular events, or whether [^18^F] NaF uptake on one side more strongly correlates with these long-term outcomes. Additionally, as previously proposed by Johnsrud et al., high inter-reader agreement is necessary for the practical clinical use of [^18^F] NaF PET/CT in the assessment of plaque progression [50]. Lastly, our study lacks histological data that could further validate the presence or absence of calcification in the studied ROIs. 

From a technical perspective, one major constraint of our method is the spatial resolution inherent to PET. Our quantitative analysis did not account for the partial volume effect, which might have impacted our findings. The LCC wall’s dimensions are smaller than the PET’s spatial resolution; nonetheless, this anatomical site is largely unaffected by movements associated with the cardiac and respiratory cycles, mitigating potential partial volume errors. Additionally, the uptake of [^18^F] NaF was assessed through a global evaluation, which to some extent counteracts the partial volume effect. This method is not affected by challenges in pinpointing specific lesions, offering a dependable overview of arterial wall atherosclerotic load. This is particularly relevant for our study, which primarily consisted of low-risk participants.

## 5. Conclusions

[^18^F] NaF uptake in the LCC and RCC was found to correlate with cardiovascular risk factors, including age, BMI, and fibrinogen. RCC [^18^F] NaF uptake also correlated directly with HDL cholesterol and inversely with male gender and CRP. For both carotid arteries, [^18^F] NaF uptake was significantly higher in patients at risk for CVD compared to healthy controls. Moreover, tracer uptake in both carotid arteries strongly correlated with the risk of cardiovascular events as estimated via the FRS. The findings from this study support the notion that [^18^F] NaF PET/CT is a reliable indicator of cardiovascular risk. [^18^F] NaF is sensitive and specific to active atherosclerotic microcalcification in both LCC and RCC: the uptake of this tracer was found to correlate with cardiovascular risk irrespective of carotid laterality, while also revealing lateral-specific variations in its correlation with cardiovascular risk factors. The association with cardiovascular risk profile presents a convincing case for the use of [^18^F] NaF PET/CT in the clinical identification and monitoring of patients at high risk of CVD. Further studies are needed to investigate whether [^18^F] NaF can serve as a marker of disease activity, a predictor of disease progression, and an indicator of long-term major cardiovascular adverse events. This research has the potential to significantly impact long-term health and quality of life for patients worldwide, as accurate, early identification of atherosclerosis can allow for patients to reversibly modify their disease via lifestyle modifications [51].

## Figures and Tables

**Table 1:** Baseline characteristics of the 102 patients included in the study.

Demographics	
Age, years	48.2 ± 14.1
Male gender, *n* (%)	52 (51)
Body mass index, kg/m^2^	26.5 ± 4.0
Comorbidities	
Active smoking, *n* (%)	12 (12)
Family history of coronary artery disease, *n* (%)	25 (24)
Arterial hypertension, *n* (%)	45 (44)
Hypercholesterolemia, *n* (%)	18 (17)
Diabetes mellitus type II, *n* (%)	0 (0)
Coronary artery disease, *n* (%)	N/A
Peripheral artery disease, *n* (%)	4 (4)
Chronic kidney disease, *n* (%)	N/A
History of previous stroke/transient ischemic attack, *n* (%)	2 (2)
Laboratory tests	
Total cholesterol, mmol/L	5.1 ± 0.9
HDL cholesterol, mmol/L	1.4 ± 0.4
LDL cholesterol, mmol/L	3.2 ± 0.8
Triglycerides, mmol/L	1.1 ± 0.7
HbA1c, mmol/mol	35.2 ± 4.9
C-reactive protein, mg/L	2.5 ± 3.5
White blood cell count, 10^9^ cells/L	6.1 ± 2.1
Fibrinogen, μmol/L	10.1 ± 7.4
Creatinine, μmol/L	79.9 ± 16.8
Estimated glomerular filtration rate, mL/min/1.73 m^2^	81.0 ± 14.8
Medications	
Aspirin, *n* (%)	9 (8)
Beta blockers, *n* (%)	9 (8)
Angiotensin-converting enzyme blockers/angiotensin receptor blockers, *n* (%)	13 (12)
Lipid-lowering medication, *n* (%)	14 (13)
Risk profile	
10-year Framingham risk, % (25–75th percentile)	8 (2–10)

**Table 2:** Regression analysis for determinants of left carotid artery [^18^F] NaF uptake.

	Univariable		Multivariable	
Predictor	β (95% CI)	*p*	β (95% CI)	*p*
Age	0.006 (0.003–0.01)	<0.01	0.005 (0.003–0.008)	<0.01
Male gender	–0.08 (−0.18–0.01)	0.11	–0.08 (−0.17 to −0.002)	0.04
Smoking (former or current)	0.01 (−0.14–0.17)	0.81		
Total cholesterol	0.002 (−0.05–0.06)	0.79		
HDL cholesterol	0.06 (−0.05–0.17)	0.29	0.07 (−0.01–0.16)	0.16
LDL cholesterol	–0.005 (−0.06–0.05)	0.85		
Triglycerides	–0.05 (−0.12–0.01)	0.09		
HbA1c	–0.001 (−0.01–0.009)	0.79		
CRP	–0.01 (−0.02–0.0006)	0.06	–0.01 (−0.02–0.001)	0.07
Fibrinogen	0.007 (0.0009–0.01)	0.02	0.006 (0.0009–0.01)	0.02
WBC count	–0.01 (−0.04–0.006)	0.14		
eGFR	0.001 (−0.002–0.004)	0.40		
BMI	0.02 (0.01–0.03)	<0.01	0.02 (0.01–0.03)	<0.01
Arterial hypertension	–0.08 (−0.19–0.03)	0.45		

**Table 3:** Regression analysis for determinants of right carotid artery [^18^F] NaF uptake.

	Univariable		Multivariable	
Predictor	β (95% CI)	*p*	β (95% CI)	*p*
Age	0.007 (0.004–0.01)	<0.01	0.006 (0.004–0.009)	<0.01
Male gender	–0.14 (−0.23 to −0.04)	<0.01	–0.13 (−0.21 to −0.06)	<0.01
Smoking (former or current)	–0.05 (−0.20–0.10)	0.55		
Total cholesterol	0.005 (−0.04–0.06)	0.74		
HDL cholesterol	0.11 (0.01–0.22)	0.03	0.13 (0.04–0.21)	<0.01
LDL cholesterol	–0.01 (−0.07–0.04)	0.55		
Triglycerides	–0.05 (−0.12–0.01)	0.10		
HbA1c	–0.002 (−0.01–0.007)	0.61		
CRP	–0.01 (−0.03–0.003)	0.01	–0.01 (−0.02–0.001)	0.03
Fibrinogen	0.007 (0.0006–0.01)	0.03	0.005 (0.001–0.01)	0.01
WBC count	–0.01 (−0.03–0.01)	0.40		
eGFR	0.001 (−0.001–0.004)	0.40		
BMI	0.01 (0.007–0.02)	<0.01	0.02 (0.01–0.03)	<0.01
Arterial hypertension	–0.04 (−0.15–0.06)	0.13		

## Data Availability

The data presented in this study are available on request from the corresponding author.
